# Whole exome sequencing identifies *KIF26B*, *LIFR* and *LAMC1* mutations in familial vesicoureteral reflux

**DOI:** 10.1371/journal.pone.0277524

**Published:** 2022-11-23

**Authors:** Zsuzsa I. Bartik, Ulla Sillén, Anna Djos, Anna Lindholm, Susanne Fransson

**Affiliations:** 1 Department of Paediatric Surgery, Paediatric Uronephrologic Centre, Queen Silvia Children’s Hospital, Göteborg, Sweden; 2 Institute of Clinical Sciences, Sahlgrenska Academy, University of Gothenburg, Gothenburg, Sweden; 3 Department of Laboratory Medicine, Institute of Biomedicine, Sahlgrenska Academy, University of Gothenburg, Gothenburg, Sweden; 4 Department of Paediatrics, County Hospital Ryhov, Jönköping, Sweden; University of Hyderabad, INDIA

## Abstract

Vesicoureteral reflux (VUR) is a common urological problem in children and its hereditary nature is well recognised. However, despite decades of research, the aetiological factors are poorly understood and the genetic background has been elucidated in only a minority of cases. To explore the molecular aetiology of primary hereditary VUR, we performed whole-exome sequencing in 13 large families with at least three affected cases. A large proportion of our study cohort had congenital renal hypodysplasia in addition to VUR. This high-throughput screening revealed 23 deleterious heterozygous variants in 19 candidate genes associated with VUR or nephrogenesis. Sanger sequencing and segregation analysis in the entire families confirmed the following findings in three genes in three families: frameshift *LAMC1* variant and missense variants of *KIF26B* and *LIFR* genes. Rare variants were also found in *SALL1*, *ROBO2* and *UPK3A*. These gene variants were present in individual cases but did not segregate with disease in families. In all, we demonstrate a likely causal gene variant in 23% of the families. Whole-exome sequencing technology in combination with a segregation study of the whole family is a useful tool when it comes to understanding pathogenesis and improving molecular diagnostics of this highly heterogeneous malformation.

## Introduction

Primary vesicoureteral reflux (VUR) is a congenital urinary tract defect that occurs in approximately 1 to 2% of young children [1]. High-grade VUR in infants is often associated with congenital generalised kidney damage, renal hypodysplasia, whereas the commonly seen acquired focal scarring is caused by ascending urinary tract infections (UTI) [2]. The morbidity seen in children with VUR is often related to recurrent UTI, with the risk of progressive kidney damage. There is an initial difference in gender prevalence in VUR in infants. Initially it affects mainly boys but there is a decline in the male-to-female ratio over time, with similar occurrences in boys and girls by the age of two [3].

Familial clustering of VUR is well recognised, indicating a strong genetic contribution to the pathogenesis. The risk that offspring will have reflux has been reported to be as high as 66%, while it is 27 to 51% for siblings [4–7]. The high frequency of VUR in relatives favours an autosomal dominant inheritance pattern with reduced penetrance [8–12], although some authors favour a possible recessive [13] or X-linked model [14]. Despite the apparent Mendelian inheritance pattern seen in many families, only a few causal genes such as *EYA1*, *PAX2*, *RET*, *ROBO2* and *SALL1* have been identified so far [12, 15–17]. However, a large number of additional candidate genes have been suggested as contributing to VUR aetiology and these mainly includes genes functioning in pathways involved in the development of the kidney, ureter and ureterovesical junction (UVJ). The two major embryological structures are the ureteric bud (UB), a budding on the metanephric duct, and the metanephric mesenchyme (MM) which is invaded by the UB and initiate branching [18]. The UVJ, ureter, renal pelvis and collecting ducts have been shown to originate from UB epithelial cells, whereas the epithelium in the nephrons (tubuli and glomeruli) originates from MM through mesenchymal-epithelial transition (MET) [19]. Interference in the interaction between the UB and the MM can result in both renal parenchymal dysgenesis and urinary tract malformation. To emphasise this association, the term CAKUT (congenital anomalies of the kidney and urinary tract) was coined [20]. Embryological work in mice has shown that many genes are involved in these developmental processes, including *Eya1*, *Pax2*, *Agtr2*, *Bmp4*, *Gdnf*, *Ret*, *Wnt11*, *Foxc1*, *Sall1*, *Robo2*, *Slit2*, *Gata3*, *Fgfr2*, *Upk2*, *Upk3 and Six1* [19–21]. Nevertheless, the entire repertoire of relevant genes is still unknown. The experimental models also suggest that a mutation affecting a single gene may result in different phenotypes, while mutations of different genes can result in the same disease [21].

In humans, different strategies have been used over the past few decades to elucidate the genetic background of primary nonsyndromic VUR. These include gene expression studies [22], association-, linkage- and exon-sequencing studies of candidate genes [23–27], genome-wide linkage and association studies [1, 9, 13, 28–33] and array-based comparative genomic hybridisation [34]. In recent years, next-generation sequencing has revolutionised genomic research. Whole-exome sequencing (WES) provides rapid detection of DNA variants within the coding part of the genome and an opportunity to arrive at a molecular diagnosis with a single test. These recent studies using WES analysis detected various variants in candidate genes in 3.2% to 17.6% of patients with CAKUT, including VUR [35–40]. The variety of candidate genes and possible loci that have been suggested in these previous studies implies that VUR is a genetically heterogeneous disease with mutations in different genes, each accounting for a proportion of cases [13]. However, WES, has limited capacity to detect structural variants, smaller copy number changes or aberrations in regulatory regions, meaning that additional causative genetic alterations could be missed. Once we have discovered the genetic background of VUR, mutation analyses of blood samples or buccal smears may replace voiding cystourethrogram (VCUG) as a screening method for relatives of VUR patients. Furthermore, these analyses will hopefully identify patients at risk by distinguishing severe cases that require prompt treatment and frequent follow-up from those where the disease is relatively benign and may resolve spontaneously. In the present study, our aim was to identify likely disease-causing gene variants in familial primary nonsyndromic VUR, focusing on patients with the infantile form of high-grade reflux and with congenital kidney hypodysplasia as we hypothesise that congenital cases are more likely to have a genetic component than cases with kidney damage due to multiple UTI. Thirteen large families with three or more affected cases were analysed by WES, focusing on genes previously established as having links to VUR as well as other candidate genes associated with embryological development of the kidney. The questions were whether one candidate gene causes the disease in all or some of the families or, if this is not the case, whether members of a family all share the same variant of a candidate gene.

## Materials and methods

### Patients and families

Thirteen families with three or more members with primary VUR were recruited at Queen Silvia Children’s Hospital (a tertiary referral centre) in Gothenburg, Sweden. All recruited families were from the south-western region and of Swedish ancestry. The families were contacted and given verbal and written information about the study. Before entering the study, all subjects and/or their parents signed an informed consent for genetic screening. Individuals older than 18 years of age signed the consent themselves. For minors written consent was obtained from both guardians. The Regional Ethical Review Board in Gothenburg approved the study (Dnr 589–05). All methods were carried out in accordance with relevant guidelines and regulations including the Declaration of Helsinki.

**Fig 1:**
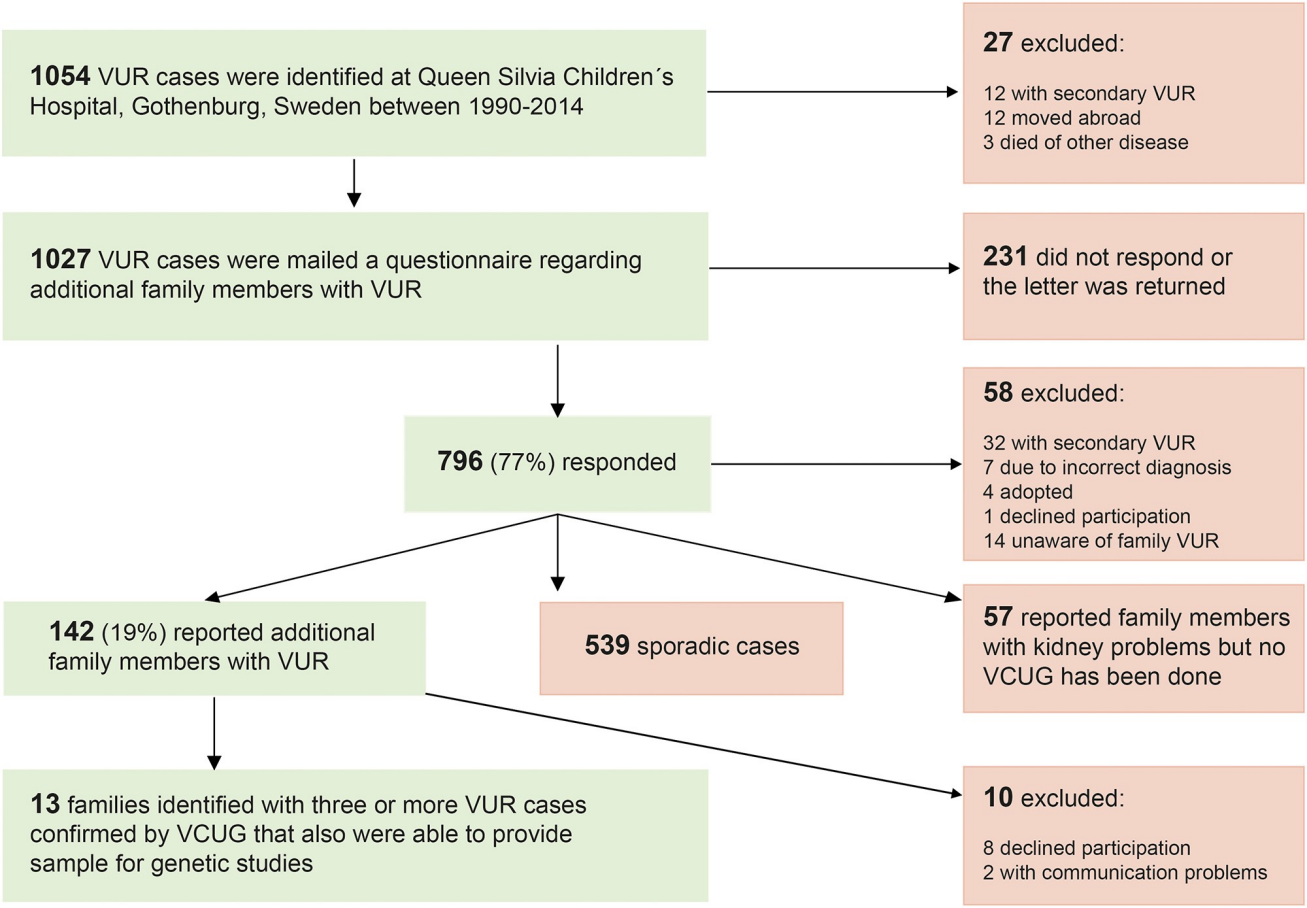
Data selection process for the study.

Blood samples or buccal swab specimens were collected by standard procedures. For the individuals in the families selected for WES, blood sampling was mandatory. Seven of these families had already participated in our previous study of hereditary VUR [27]. The selection process for the study, with initial cases and with subsequent inclusion and exclusion criteria, is presented in Fig 1.

Clinical data was obtained from medical records, VUR grade from voiding cystourethrograms (VCUG), permanent kidney damage from scintigraphy with Tc-99m dimercaptosuccinic acid (DMSA) or Tc-99m mercaptoacetyltriglycine (MAG3) and total kidney function from glomerular filtration rate (GFR) measurements or by estimations following the Schwartz formula [41, 42]. In the case of bilateral VUR, the patient was classified according to the more severely affected side in terms both of VUR grade and kidney damage. Focal kidney damage was defined as one or more areas with reduced uptake or indentation of the kidney outline caused by postnatally acquired kidney scarring [2]. Generalised damage was defined as a small kidney with reduced tracer uptake or a diffuse parenchymal anomaly, referred to as congenital renal hypodysplasia [43, 44]. A GFR of < 80% (<2SD) of expected GFR was considered subnormal. GFR reference values in children under two years of age were calculated using Winberg’s algorithm [45]. For older children a rate of 110 ml/min/m^2^ was used.

**Fig 2:**
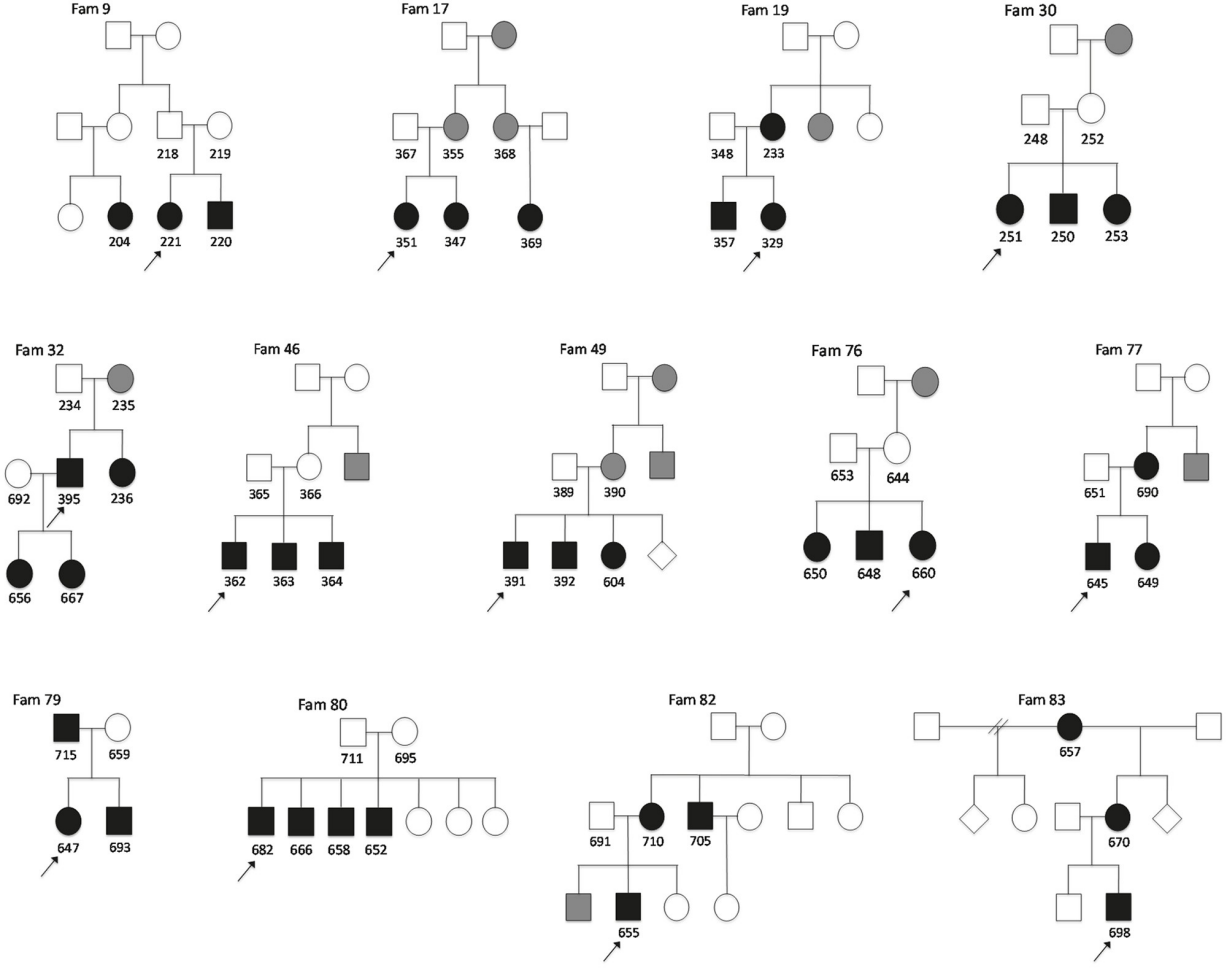
Pedigrees of included families. Pedigrees describe the 13 participating families with three or more vesicoureteral reflux cases. A family identifier is indicated above each respective pedigree with case-specific identifiers given under each individual. *Squares* males, *circles* females, *rhombuses* sex unknown, *black symbols* indicate diagnosis confirmed by voiding cystourethrography, *grey symbols* indicate strong history of VUR but no available radiological investigations, *arrows* index cases.

To clarify the relationship and analyse the pattern of inheritance, pedigrees were constructed for each family (Fig 2). Additional members of families with a history strongly suggesting VUR but with no radiological test results, were classified as probable cases. Patients with secondary VUR, e.g., patients with neurogenic bladder or posterior urethral valves, were excluded from the study.

### Whole-exome sequencing

The most severely affected family member, meaning a member with confirmed generalised kidney damage (renal hypodysplasia), was chosen for WES. When this was not possible, the selection criterion used was high-grade VUR. In three families, WES was carried out on an additional individual. What the three additional study subjects had in common was that they were the most distantly-related, affected relatives of the proband in their respective pedigree (aunt, uncle and cousin).

**Fig 3:**
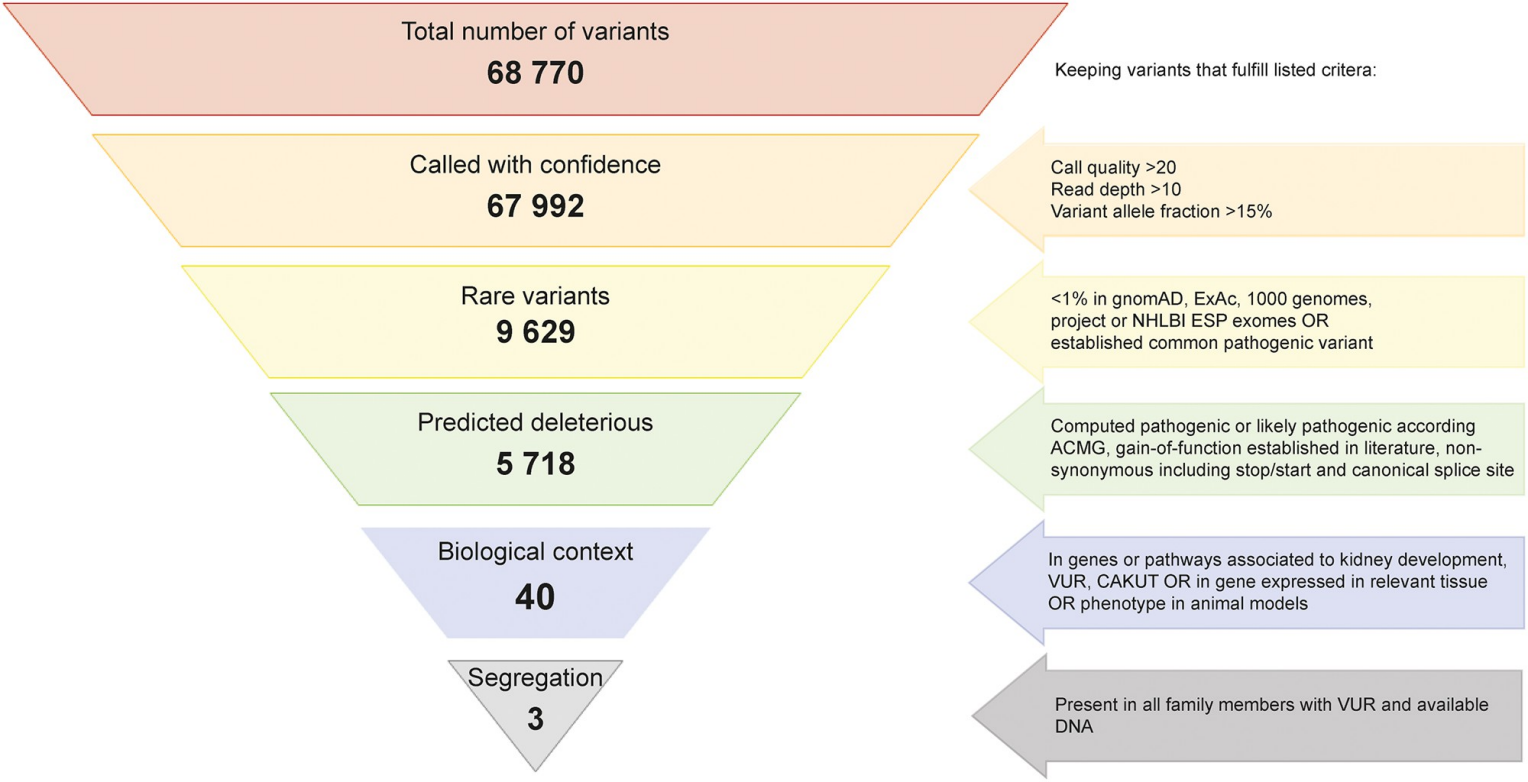
Filtering of called variants. Systematic filtering of single nucleotide variants called from CLC workbench with number of variants remaining after each step and criteria for retaining variant as indicated.

Genomic DNA was isolated from blood lymphocytes and subjected to WES (GATC, Constance, Germany) on Illumina instrumentation (Illumina, San Diego, CA) after DNA enrichment using Agilent SureSelect human All exon v6 (Agilent technologies, Santa Clara, CA) reaching an average coverage of 70X (range 46-114X). Coverage and mapping metrics are presented in S1 Table. Read trimming, mapping, and variant calling were performed using CLC Biomedical Genomics Workbench software (Qiagen, Aarhus, Denmark) (S1 File) with consecutive variant filtering using Qiagen QCI interpret translational tool (Qiagen). Only high-quality called variants with a variant allele frequency above 0.15 and a total read coverage of at least ten were considered for further analysis. Variants with a minor allele frequency above 0.01 in either SweGen dataset (https://swegen-exac.nbis.se), 1000 genomes, Exome Aggregation Consortium (ExAC), Cambridge, MA (http://exac.broadinstitute.org), Genome Aggregation Database (gnomAD) http://gnomad.broadinstitute.org or NHLBI Exome Sequencing Project (http://evs.gs.washington.edu/EVS/) were discarded, as well as all synonymous variants or variants in non-coding regions, except those affecting canonical splice sites. Remaining variants were assessed manually through the Integrative Genomics Viewer (IGV) [46]. PolyPhen 2, SIFT and CADD were used to predict the functional relevance of called single nucleotide variants (SNV)s. Possible relevance to the biological disease context was assessed using QCI interpret translational (Qiagen). The filtering process and remaining variants after each step are visualized in Fig 3. All genomic positions were given according to the human reference genome GRCh37/hg19.

### Variant classification and prioritisation

The remaining potential rare causal gene variants were further filtered in an extensive literature search. This literature review focused on: 1) gene function and associated phenotype, 2) gene-associated animal models, 3) tissue expression of the encoded protein, 4) association with already known VUR genes and 5) location of the variant with respect to functional protein domains. Genes participating in Ureteric Bud/Metanephric Mesenchyme development were regarded as highly relevant. Genes associated with syndromes were included if a connection to kidney development or VUR was stated. Syndromes with other CAKUT phenotypes were excluded.

Our different strategies for prioritising the findings were: 1) screening for variants of genes previously associated with VUR and kidney development, 2) screening for common variants in different families and 3) screening for common variants within the family in the three applicable cases.

### Sanger sequencing

Sanger sequencing was used to verify significant WES findings in subjects, as well as for segregation analysis of all healthy and sick relatives where samples were available. DNA was extracted using a Maxwell 16 Buccal Swab LEV DNA Purification Kit (Promega, Madison, WI) for samples collected with Isohelix buccal swabs, while Qiagen DNeasy Blood & Tissue Kit (Qiagen, Aarhus, Denmark) was used for blood. ExonPrimer (https://ihg.helmholtz-muenchen.de/ihg/ExonPrimer.html) was used to design primers. Primer sequences and other PCR details are available upon request. Sanger sequencing was performed by GATC Biotech (Constance, Germany) and analysed using the SnapGene software (GSL Biotech, Chicago, IL).

## Results

### Clinical characteristics

A total of 41 patients from 13 different families with VUR were included (20 males, 21 females), of whom 16 were subjects for WES. There were two nuclear families and 11 extended families. The relationship between the affected individuals and the pattern of inheritance is shown in Fig 2 and S2 Table.

Demographics and phenotypic details of the study subjects are outlined in Table 1. The whole-exome sequenced study subjects were more commonly male (62%), with a higher grade of reflux (69% grade IV-V in the sequenced cohort vs. 49% in the whole group), with more generalised kidney damage (81% vs. 53%) and frequently subnormal total kidney function (25% vs. 13%). Only five cases showed additional malformations of the urinary tract, such as bilateral duplex kidney (1), bladder diverticula (3) and unilateral megaureter (1). In addition, three cases with extrarenal manifestations had syndromic features but did not have a known diagnosis (S3 Table).

**Table 1:** Demographic data, VUR grades, kidney abnormalities and function for the whole-exome sequenced group and for the whole study group.

Characteristics	Values WES cohort n = 16	Values all VUR cohort n = 41
Gender		
Female	6 (38%)	21 (51%)
Male	10 (62%)	20 (49%)
Presenting symptom VUR		
Pyelonephritis	11 (69%)	28 (68%)
Pre and postnatal screening	4 (25%)	10 (25%)
Other symptoms	1 (6%)	3 (7%)
Age at presentation (months)	7 (0.25–98)	7 (0.25–98)
Grade of reflux		
I–III	5 (31%)	21 (51%)
IV–V	11 (69%)	20 (49%)
Uni or bilateral reflux		
Unilateral	5 (31%)	16 (39%)
Bilateral	11 (69%)	25 (61%)
Recurrent UTIs		
No	5 (33%)	13 (33%)
Yes	10 (67%)	26 (67%)
Kidney damage		
No	2 (13%)	14 (35%)
Yes, focal	1 (6%)	5 (12%)
Yes, generalised *	13 (81%)	21 (53%)
Uni or bilateral kidney damage		
Unilateral	10 (71%)	21 (81%)
Bilateral	4 (29%)	5 (19%)
Total kidney function		
Normal	12 (75%)	32 (84%)
Subnormal	4 (25%)	6 (16%)

Categorical variables n (%), Continuous variables median (range),

*Hypodysplasia

### Candidate variants in VUR/nephrogenesis genes

We performed WES on 16 individuals from 13 families with hereditary VUR and after multistep variant filtering and prioritisation, as described in Materials and Methods, 40 heterozygous candidate variants in 32 genes previously associated with VUR or nephrogenesis were retained (S4 Table). They included variants in genes previously associated with diseases showing autosomal recessive inheritance such as *FREM2*, *ROR2* and *FRAS1* although none of them were homozygous or compound heterozygous.

To further elucidate whether additional members within the same family had inherited the same variant, WES was performed on a second affected member in three families (see Material and methods). In one family (Fam. 32) with severe VUR and renal hypodysplasia, two DNA variants in possible causal genes (*LIFR*, *CLDN3*) were detected in both patients while in the second family (Fam. 17), a novel *KIF26B* variant was shared by the two family members who had been investigated (Table 2, Fig 2). The third family (Fam. 82) did not share any variant in kidney-associated genes, in spite of their astonishingly similar phenotype with explicit generalised kidney damage.

**Table 2:** Results of Sanger sequencing used for segregation analysis in 13 families with hereditary VUR.

Family	Genes	Protein change	Investigated (WES)		Investigated (Sanger sequencing)				
**9**			**221 F**		**220 M**	**204 F**	218^c^M	219 F	
	*SALL2*	p.P168L	**+**		**+**	**-**	**+**	**-**	
	*SIM1*	p.G254K	**+**		**+**	**-**	**-**	**-**	
**17**			**351 F**	**369 F**	**347 F**	**355** ^p^ **F**	**368** ^p^ **F**	367 M	
	***KIF26B***	p.S123L	**+**	**+**	**+**	**+**	**+**	**-**	
	*UPK2*	splice site loss	**+**	**-**	**+**	**+**	**-**	**-**	
**19**			**357 M**		**329 F**	**233 F**	348 M		
	*SALL1*	p.G1168E	**+**			**-**	**+**		
	***CHD7***	p.L935F	**+**		**+**	**+**	**-**		
	*LIFR*	p.D816G	**+**		**-**	**+**	**-**		
**30**			**250 M**		**251 F**	**253 F**	252^c^F	248 M	
	*MDM4*	p.K374Q	**+**		**-**	**+**	**+**	**-**	
	*CLDN3*	p.P134L	**+**		**+**	**+**	**-**	Hom	
	*SALL2*	p.T45N	**+**		**-**	**+**	**-**	**+**	
**32**			**236 F**	**656 F**	**395 M**	**235** ^p^ **F**	234 M		
	***LIFR***	p.V487A	**+**	**+**	**+**	**+**	**-**		
	***CLDN3***	p.P134L	**+**	**+**	**+**	**+**	**-**		
	*GLI3*	p.R114K	**-**	**+**		**-**	**-**		
	*CHD7*	p.L935F	**-**	**+**	**-**	**-**	**-**		
**46**			**364 M**		**362 M**	**363 M**	366^c^F	365 M	
	***MMP9***	p.R24C	**+**		**+**	**+**	Hom	**-**	
	*SALL2*	p.P168L	**+**		**-**	**+**	**-**	**+**	
	*TGFBR3*	p.F434S	**+**		**-**	**-**	**-**	**+**	
**49**			**391 M**		**392 M**	390^c^F	389 M		
	*GATA3*	p.P154S	**+**		**-**	**+**	**-**		
	*PYGO1*	p.N250I	**+**		**-**	**+**	**-**		
**76**			**650 F**		**648 M**	**660 F**	644^c^F	653 M	
	*ROBO2*	p.I598T	**+**		**-**	**+**	**-**	**+**	
	*FRAS1*	p.M2129V	**+**		**-**	**-**	**-**	**+**	
	***LAMC1***	p.K646fs*3	**+**		**+**	**+**	**+**	**-**	
	***GREB1L***	p.E93K	**+**		**+**	**+**	**+**	**-**	
**77**			**645 M**		**649 F**	**690 F**	651 M		
	*BMP7*	p.N321S	**+**		**-**	**-**			
	*WNT3A*	p.A172T	**+**			**-**			
	*POSTN*	p.Q71K	**+**			**-**			
	*KIF26B*	p.S1218F	**+**		**-**	**+**	**-**		
**79**			**715 M**		**647 F**	**693 M**	659 F		
	*FRAS1*	p.Y1758C	**+**		**+**	**-**	**-**		
	*NRTN*	p.V125L	**+**						
	*TGFBR3*	p.P776S	**+**		**+**	**?**	**-**		
**80**			**682 M**		**652 M**	**658 M**	**666 M**	695 F	711 M
	*SLIT3*	p.S629N	**+**		**-**	**+**	**-**	**+**	**-**
**82**			**655 M**	**705 F**	**710 F**	691 M			
	*UPK3A*	p.W182*	**+**	**-**	**-**	**+**			
	*CHD1L*	p.G491R	**+**	**-**					
	*MMP9*	p.R24C	**-**	**+**	**-**	**-**			
	*TGFBR3*	p.H155R	**-**	**+**	**-**	**-**			
**83**			**698 M**		**670 F**	**657 F**			
	*DSTYK*	splice site loss	**+**		**+**	**-**			
	*MDM4*	p.K374Q	**+**		**-**	**-**			
	*GREB1L*	p.E93K	**+**		**-**	**-**			

**bold digit**, affected family members; **bold gene symbol**, the gene variant segregates with the phenotype in the family; F, female; hom, homozygous variant; M, male; **+**, variant present in heterozygous form; **-**, variant missing; ?, Sanger sequencing failed, chromatogram not assessable

^c^ Probable carrier according to the pedigree

^p^ Probable VUR, strong history of VUR but no available radiological investigations.

**Fig 4:**
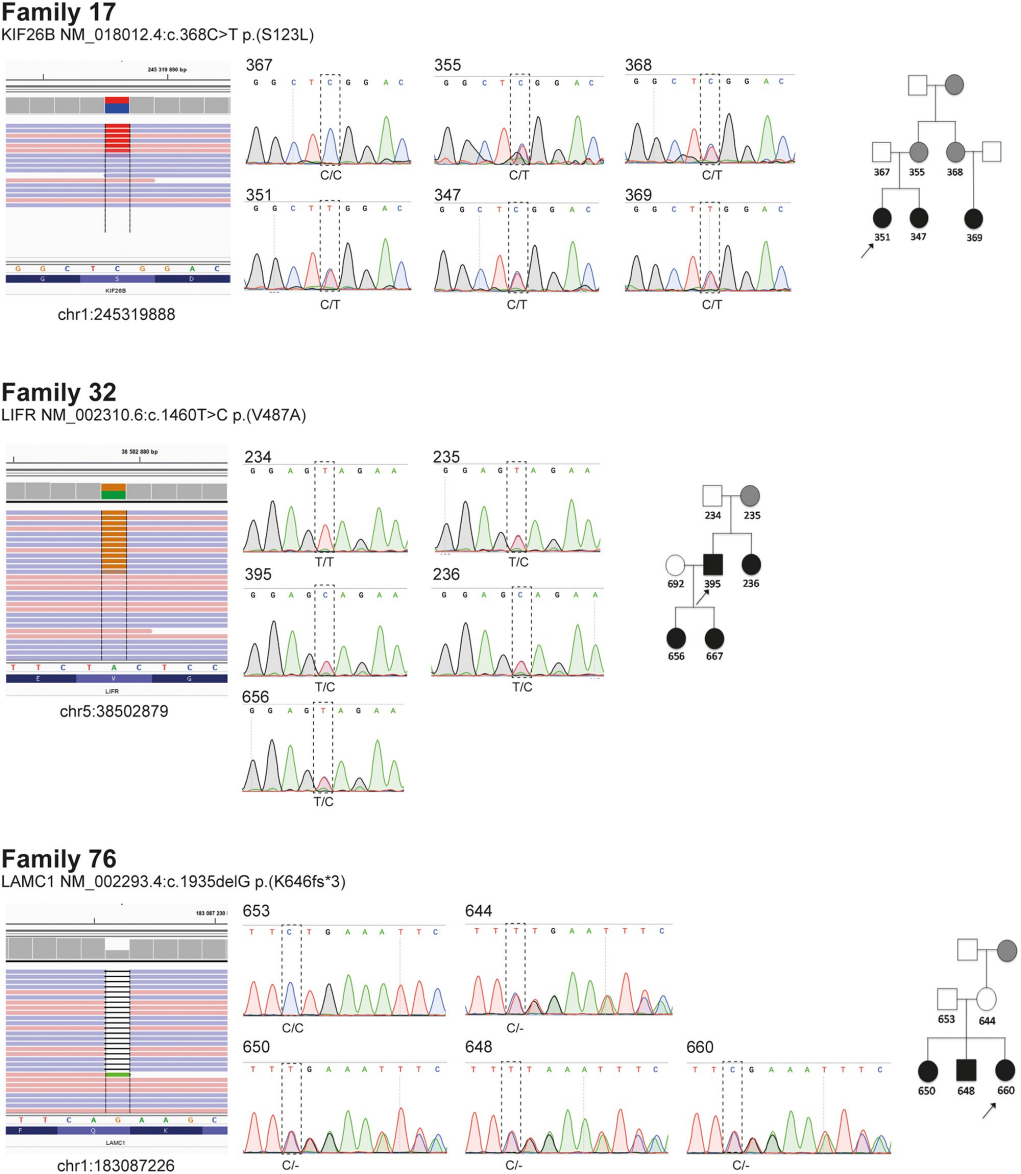
Variant identification and segregation analysis. Candidate variants detected by WES visualized in IGV with genomic position as indicated (left panels). Electropherograms from Sanger sequencing over corresponding positions show that the variants in *KIF26B* (fam. 17), *LIFR* (fam. 32) and *LAMC1* (fam. 76) segregate with disease in respective families.

A segregation analysis was performed on all candidate variants (except the genes with autosomal recessive inheritance lacking biallelic alterations, as judged from WES) in all relatives with available DNA samples. Sanger sequencing showed variants segregating with disease in three different families (Table 3, Fig 4). This was in three nephrogenesis-related genes (*KIF26B*, *LAMC1* and *LIFR*) in which autosomal dominant inheritance had previously been reported. Despite being highly interesting in regard to VUR aetiology, the remaining variants which were analysed did not segregate with the phenotype in all the families concerned (Table 2, S4 Table).

**Table 3:** Three possibly pathogenic variants identified in nephrogenesis-related genes in three families with hereditary VUR.

Gene	Family-Individual	Renal phenotype	Extrarenal phenotype	Variants^a^	MAF SweGene	MAF gnomAD	Impact	SIFT^b^	PP2^c^	Reference
*KIF26B*	17–351	B VUR, U FRD	Scoliosis, MI, JIA, Marfan?	NM_018012.4:c.368C>T p.(S123L)	Novel	Novel	M	D	0.952	16, 53–55
	17–369	U VUR		NM_018012.4:c.368C>T p.(S123L)						
*LAMC1*	76–650	B VUR, U RHD		NM_002293.4:c.1935delG p.(K646fs*3)	Novel	0.000004	F	NA	NA	16, 40, 51, 52
*LIFR*	32–656	B VUR, U RHD, SubnRF		NM_002310.6:c.1460T>C p.(V487A)	Novel	Novel	M	A	0	38, 56, 57
	32–236	U VUR, U RHD		NM_002310.6:c.1460T>C p.(V487A)						

Abbreviations; A, activating; B, bilateral; D, damaging; F, frameshift; FRD, focal kidney damage; JIA, juvenile idiopathic arthritis; M, missense; MI, mitral insufficiency; NA, no prediction available; RHD, renal hypodysplasia; SubnRF, subnormal total kidney function; T, tolerated; U, unilateral; VUR, vesicoureteral reflux.

^a^, All mutations are heterozygous;

^b^, Sorting Intolerant From Tolerant (http://sift.bii.a-star.edu.sg);

^c^, PolyPhen-2 prediction score ranges from 0 (= benign) to 1 (= probably damaging) (http://genetics.bwh.harvard.edu/pph2/)

Predicted deleterious or truncating variants that did not show consistent co-occurrence with a VUR phenotype included predicted deleterious, missense variants in the known VUR genes, *SALL1* (Fam. 19), *ROBO2* (Fam. 76), and *UPK3A* (Fam. 82). These were inherited from healthy fathers in the families while splice site variants in *UPK2* (Fam. 17) and *DSTYK* (Fam. 83) were present in some but not all affected family members (Table 2). Variants in *GREB1L* and *CLDN3* segregated with disease in Family 76 and Family 32 respectively. However, both variants were also detected in other families in the study cohort: the *GREB1L* was also detected in the youngest individual in Family 83, but was not seen in other affected relatives while the *CLDN3* variant was also present in homozygous form in the unaffected father in Family 30.

One *MMP9* variant was detected in affected individuals in Families 46 and 82, although only segregating in Family 46, where one unaffected family member (individual 366) was homozygous for the variant. Similarly, a variant in *CHD7* was detected in two families (Fam. 19 and Fam. 32), but it segregated only in Family 19. None of the study subjects carrying this *CHD7* allele showed the syndromic phenotype described in the literature on this gene, indicating that this variant is most likely benign.

### Signalling pathways in the embryological development of the kidney

The three genes with potential pathogenic variants in families with VUR and kidney damage participate in different signalling pathways that are crucial for the development of the lower urinary tract and kidney. They include mitogen-activated protein kinase (MAPK) (genes: *KIF26B*, *LIFR*), Wnt (genes: *KIF26B*, *LAMC1*, *LIFR*), phosphoinositide 3-kinase (PI3K)/AKT (genes: *LAMC1*, *LIFR*, *KIF26B*) and Janus kinase/signal transducers and activators of transcription (JAK/STAT) (gene: *LIFR*). A simplified diagram of the interactions between the genes (including our initially most promising candidate genes) is shown in S2 File. Due to the interdependence between developmental pathways, mutations in different genes can result in similar phenotypes.

## Discussion

A cohort consisting of 13 large families, which originated from the west coast of Sweden and three or more of whose members had primary VUR, was investigated by WES, focusing on genes with known pathogenicity in VUR. Additional candidate genes not previously reported in patients with VUR or other CAKUT (such as *CLDN3*, *KCP*, *LAMC1*, *POSTN* and *WNT3A*) but where experimental models demonstrated expression and/or effect on UB outgrowth and tubular growth [47–50], were also included. Among these, 40 heterozygous novel or rare variants were detected in 32 different genes affecting kidney development (S4 Table). The segregation with the disease phenotype within families was ascertained by Sanger sequencing, validating three different variants affecting *LAMC1*, *KIF26B*, and *LIFR* as possible causes of VUR in three of the 13 families (Fig 4).

Among the new candidate genes, an extremely rare frameshift variant in *LAMC1* (Laminin Subunit Gamma 1) was found to segregate with VUR in Family 76. In an early study of laminins in kidney development, no phenotypic effect on the kidney was observed in mice with a heterozygous *Lamc1* mutation whereas homozygous mice died, having ectopic ureters and an absence of kidneys [51]. *Lamc1* was found to regulate branching morphogenesis where inactivation of *Lamc1* in the UB resulted in small kidneys or absence of kidneys, and ureters with empty bladders [52]. Although it is not clear if heterozygous mutations in *LAMC1* could affect the kidney phenotype in humans, it is believed that there is a laminin concentration threshold above which UB penetration is enabled, determining the development of renal hypodysplasia or kidney agenesis [52]. In line with this, deleterious heterozygous variants in *LAMC1* have been reported in rare cases in two previous studies of CAKUT in patients with ureteropelvic junction obstruction or duplex collecting system [16, 40].

The two missense variants, both predicted to be damaging, were detected in *KIF26B* (Kinesin Family Member 26B) in Families 17 and 77 respectively. However, only the novel variant *KIF26B*^S123L^ segregated with phenotype in individual members of the family who were tested. *KIF26B* regulates the adhesion of mesenchymal cells in contact with ureteric buds and it is thus essential for the UB invasion of MM and UB branching [53]. Variants in *KIF26B* have been previously described in patients with renal hypodysplasia [54], renal coloboma syndrome [55] and multicystic dysplastic kidney [16]. The third heterozygous missense variant segregating with high-grade VUR and unilateral renal hypodysplasia was identified in *LIFR* (Leukemia Inhibitory Factor Receptor) in Family 32. *LIFR* encodes a receptor in the MM that promotes MET when bound to its ligand, LIF, secreted by the UB [56, 57]. Kosfeld et al. recently demonstrated heterozygous *LIFR* variants in 3.3% of CAKUT patients and similar anomalies in *Lifr*-deficient mice [38].

From this, a probable cause of the malformation is identified in 23% of the families in this cohort. Recent sequencing studies presented pathogenic/likely pathogenic gene variants in a smaller fraction of cases (3.2 to 17.6%) [15, 16, 37, 58]. One explanation is that their studies were on mainly non-hereditary cases with a primary focus on CAKUT rather than the VUR/renal hypodysplasia complex. Our families all had three or more individuals with the disease phenotype, in this case VUR. However, despite compelling support for a strong hereditary component, the lack of causative variants in the majority of the families and individuals in our and other studies indicates a more complex VUR aetiology. VUR appears to be a complex polygenic disorder, where a combination of risk alleles as well as environmental factors results in the disease phenotype. Kidney and ureteric development are delicate processes for which tempospatial precision is instrumental and they also involve a considerable network of proteins (partly presented in S2 File). This contributes to great heterogeneity among genes and gene variants, which could cause disease where dysfunctional. In line with this, we detect rare, damaging variants that do not segregate fully with disease within the family. These include variants in *GREB1L*, *UPK2*, *DSTYK* and *SLIT3*, all genes which have previously been associated with impaired ureteric and kidney development. *GREB1L*, for which a missense variant was detected in affected members of Family 76 and Family 83, encodes a cofactor in the retinoic acid mediated signalling that regulates RET expression in the UB [59]. Heterozygous knockout of *Greb1l* in mice causes a decrease in ureteric bud branching while the heterozygous *GREB1L* mutation is common in patients with renal hypodysplasia and kidney agenesis [39, 59–61]. In Family 17, which also displayed a *KIF26B* variant, the siblings and mother, but not the cousin or the aunt, had a very rare heterozygous *UPK2* splice site variant (S3 Table, Fig 2). Nicolaou et al. identified a different *UPK2* splice site variant in a patient with a duplex collecting system [16]. A splice site loss in *DSTYK* was seen in the child and mother but not the grandmother in Family 83. The same variant was identified in a large Italian family with CAKUT (where some cases had VUR) and among an additional 311 unrelated patients with CAKUT, where 2.3% displayed different *DSTYK* variants [62]. However, the pathogenicity could be disputed as a study presented the detected splice variant in a patient with suspected branchio-oto-renal syndrome but with a normal kidney ultrasound but also in 10/425 in-house controls [58], i.e. much higher than available population datasets (MAF SweGen = 0.0005, gnomAD = 0.0003).

Pathogenic or likely pathogenic variants in *EYA1*, *HNF1B*, *RET* and *PAX2* have been identified in several extensive genetic screenings of CAKUT with VUR [15, 16, 58]. However, no alterations of these genes were detected in our cohort. Instead, novel or rare variants in *KIF26B*, *LAMC1* and *LIFR*, genes associated with kidney development, were shown to segregate with disease in three out of 13 families with hereditary VUR. The *LAMC1* frameshift is likely to result in a variant causing a loss of function, while functional predictions for *KIF26B* and *LIFR* indicate damaging as well as activating effects on protein. Whereas constraint scores calculated by Lek et al. [63] indicates that both *KIF26B* and *LAMC1* are sensitive to mutations and thereby support pathogenicity, this score also indicates that *LIFR* is relatively insensitive (S4 Table). Ultimately, the degree of pathogenicity of these variants requires further functional studies of their impact on embryonic development.

One of the methodological limitations of the study is that genetic testing was not performed on all study subjects diagnosed with the disease. The study was performed partly under financial constraints. Therefore, the most severely affected sibling and, when available, an affected second-degree relative were tested, producing maximum information per test. In addition, blood samples were not available or were not available in substantial amounts from all family members. Although most people were positive to the study when they received the invitation to participate, we had recruitment problems when they were asked to donate blood samples. Using buccal smear kits sent home by post minimized the inconvenience for children and their families, and increased the willingness to participate. However, in clinical settings this method yielded DNA of suboptimal quantity and quality, insufficient for whole-exome sequencing. Finally, as VUR is a non-visible malformation in asymptomatic individuals and is sometimes spontaneously and naturally resolved during childhood, reflux is a difficult abnormality to study in terms of inheritance. VCUG is the gold standard method of detecting VUR. However, it is a highly invasive investigation, which limits its use in asymptomatic relatives, and it was not available for older family members prior to the 1960s.

In summary, the diversity of our findings together with previous studies supports the hypothesis that primary VUR from the perspective of genetics is a very heterogeneous disease, making the genetic study of familial VUR challenging. The paucity of recurrent genes with protein-changing variants could also indicate alterations in regulatory elements affecting key genes during the embryonic development of the urinary tract.

## Supporting information

S1 File Workflow overview and specific settings for bioinformatical handling of sequence data in CLC genomic workbench. (PDF)

S2 File Gene interactions and corresponding pathways in kidney development. A simplified diagram of the interactions between kidney genes with novel or rare variants detected by WES in this study. These genes, with mainly damaging, but some tolerated mutations, participate in different signalling pathways that are crucial for the development of the lower urinary tract and kidney. *Arrow*, activation; *continuous line*, direct effect; *interrupted line* indirect effect, ------I inhibition. Brief explanation of gene interactions with inclusion of selected references.
(PDF)

S1 Table Coverage and mapping metrics. (XLSX)

S2 Table Thirteen families with hereditary VUR; relationship between 13 index cases and 28 affected relatives. (XLSX)

S3 Table Additional malformations of the urinary tract (UT) and other organ systems. (XLSX)

S4 Table Intitial candidate variants after sequencing of 13 families with primary VUR. (XLSX)
