# Supplementary material for: Whole exome sequencing identifies KIF26B, LIFR and LAMC1 mutations in familial vesicoureteral reflux
Source: PLoS One. 2022 Nov 23;17(11):e0277524. doi: 10.1371/journal.pone.0277524 (PMC9683562; doi:10.1371/journal.pone.0277524)
Supplement: S1 File — (PDF) [file pone.0277524.s001.pdf]

Supplemental file 1.
CLC Genomic Workbench
workflow and settings

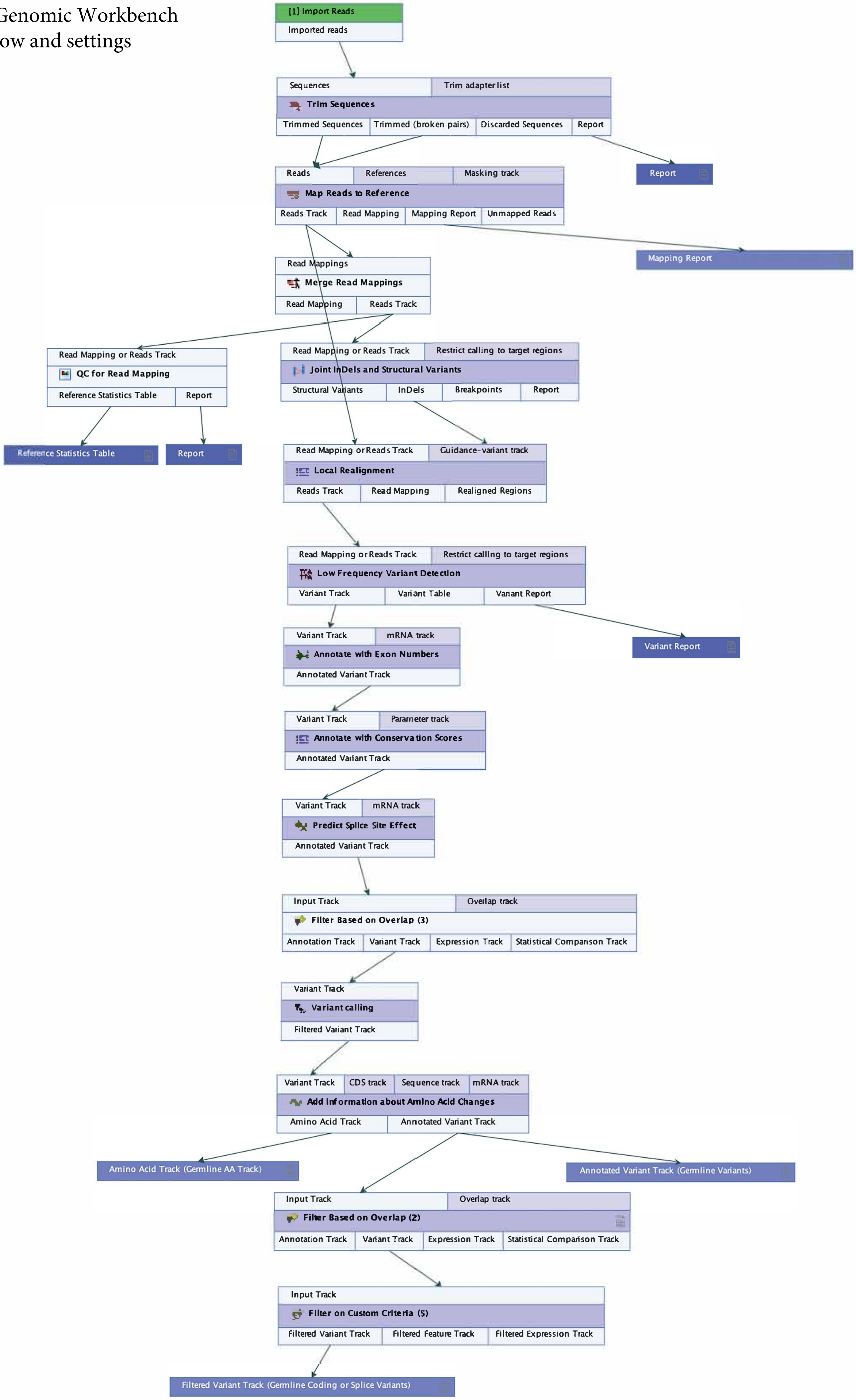

Exome - Identify and annotate

Local Realignment	
Realign unaligned ends	true
Multi-pass realignment	3
Guidance-variant track	Defined by: Joint InDels and Structural Variants
Allow guidance insertion mismatches	true
Maximum guidance-variant length	1500
Force realignment to guidance-variants	false

Low Frequency Variant Detection	
Required significance (%)	1.0
Ignore positions with coverage above	5000
Restrict calling to target regions	
Ignore broken pairs	false
Ignore non-specific matches	Reads
Minimum read length	20
Minimum coverage	10
Minimum count	3
Minimum frequency (%)	5.0
Base quality filter	true
Neighborhood radius	5
Minimum central quality	20
Minimum neighborhood quality	18
Read direction filter	true
Direction frequency (%)	5.0
Relative read direction filter	true
Significance (%)	1.0
Read position filter	false
Significance (%)	1.0
Remove pyro-error variants	true
In homopolymer regions with minimum length	8

Low Frequency Variant Detection	
With frequency below	0.5

Annotate with Exon Numbers	
mRNA track	Homo_sapiens_refseq_HG19_mRNA

Annotate with Conservation Scores	
Parameter track	PhastCons_conservation_scores_hg19

Predict Splice Site Effect	
mRNA track	Homo_sapiens_refseq_HG19_mRNA
Splice site window size	2
Discard variants without effect on splice site	false

Add Information about Amino Acid Changes (Original name: Amino Acid Changes)	
CDS track	Homo_sapiens_refseq_HG19_CDS
Sequence track	Homo_sapiens_sequence_hg19
mRNA track	Homo_sapiens_refseq_HG19_mRNA
Use transcript priorities	false
Move variants from VCF location to HGVS location	false
Include upstream flanking positions	5000
Include downstream flanking positions	2000
Filter away synonymous variants	false
Genetic code	1 Standard
Filter away CDS regions with no variants	true
Use one letter codon code	false

Variant calling (Original name: Remove Marginal Variants)	
Variant frequency	true
Minimum frequency (%)	15.0
Forward/reverse balance	false
Minimum forward/reverse balance	0.05
Average base quality	false
Minimum average base quality	20.0

Filter Based on Overlap (3) (Original name: Filter Based on Overlap)	
Overlap track	

Filter Based on Overlap (3) (Original name: Filter Based on Overlap)	
Keep overlapping	Keep annotations that do not overlap

Filter on Custom Criteria (5) (Original name: Filter on Custom Criteria)	
Criteria	any{Non-synonymous contains Yes}{ Splice Effect contains Possible}

QC for Read Mapping	
Long contigs threshold	10000
Short contigs threshold	200

Joint InDels and Structural Variants (Original name: InDels and Structural Variants)	
P-Value threshold	1.0E-4
Maximum number of mismatches	3
Minimum quality score	18
Minimum relative consensus coverage	0.0
Filter variants	true
Minimum number of reads	5
Ignore broken pairs	true
Restrict calling to target regions	

Map Reads to Reference	
References	Homo_sapiens_sequence_hg19
Masking mode	No masking
Masking track	
Match score	1
Mismatch cost	2
Cost of insertions and deletions	Affine gap cost
Insertion cost	3
Deletion cost	3
Insertion open cost	6
Insertion extend cost	1
Deletion open cost	6
Deletion extend cost	1
Length fraction	0.5
Similarity fraction	0.8
Global alignment	false
Auto-detect paired distances	true

Map Reads to Reference	
Non-specific match handling	Map randomly

Trim Sequences (Original name: Trim Reads)	
Trim using quality scores	true
Quality limit	0.02
Trim ambiguous nucleotides	true
Maximum number of ambiguities	2
Automatic read-through adapter trimming	true
Trim adapter list	
Trim homopolymers from 5'	false
Trim homopolymers from 3'	false
polyA	false
polyC	false
polyG	true
polyT	false
Remove 5' terminal nucleotides	true
Number of 5' terminal nucleotides	3
Remove 3' terminal nucleotides	false
Number of 3' terminal nucleotides	1
Remove on first read	true
Remove on second read (for paired reads)	true
Trim to a fixed length	false
Maximum length	150
Trim end	Trim from 3'-end
Discard short reads	true
Minimum length	30
Discard long reads	false
Maximum length	1000

Import Reads (Original name: Illumina High-Throughput Sequencing Import)	
Select files	
Discard read names	false
Discard quality scores	false
Paired reads	true
Read orientation	Forward Reverse
Minimum distance	1
Maximum distance	1500
Remove failed reads	true

Import Reads (Original name: Illumina High-Throughput Sequencing Import)	
Quality scores	NCBI/Sanger or Illumina Pipeline 1.8 and later
MiSeq de-multiplexing	false
Trim reads	false
Join reads from different lanes	false
Use custom reads options	false
Custom reads options	R1, R2

Filter Based on Overlap (2) (Original name: Filter Based on Overlap)	
Overlap track	Homo_sapiens_refseq_HG19_Genes
Keep overlapping	Keep annotations that overlap
