# Supplementary material for: Whole exome sequencing identifies KIF26B, LIFR and LAMC1 mutations in familial vesicoureteral reflux
Source: PLoS One. 2022 Nov 23;17(11):e0277524. doi: 10.1371/journal.pone.0277524 (PMC9683562; doi:10.1371/journal.pone.0277524)
Supplement: S2 File — A simplified diagram of the interactions between kidney genes with novel or rare variants detected by WES in this study. These genes, with mainly damaging, but some tolerated mutations, participate in different signalling pathways that are crucial for the development of the lower urinary tract and kidney. Arrow, activation; continuous line, direct effect; interrupted line indirect effect, ------I inhibition. Brief explanation of gene interactions with inclusion of selected references. (PDF) [file pone.0277524.s002.pdf]

Supplemental file 2. Bartik et al.

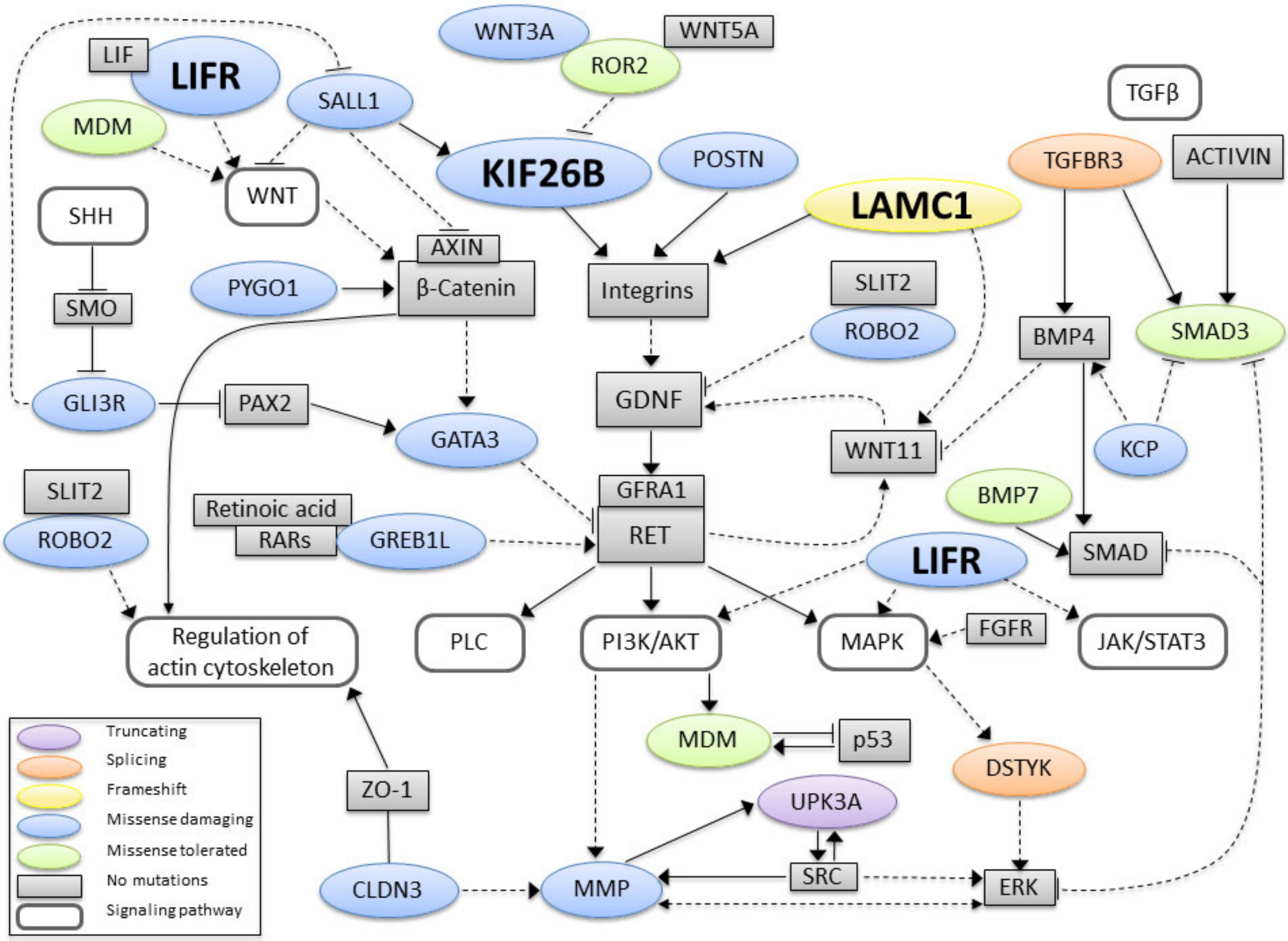

Supplemental file 2. Gene interactions and corresponding pathways in kidney development. A simplified diagram of the interactions between kidney genes with novel or rare variants detected by WES in this study. These genes, with mainly damaging, but some tolerated mutation, participate in different signalling pathways that are crucial for the development of the lower urinary tract and kidney. *Arrow,* activation; *continuous line,* direct effect; *interrupted line* indirect effect, ------I inhibition.

Gene interactions and selected references on page 2

Gene	Interactions	Reference
KIF26B	Kif26b knock out mice show decreased Gdnf expression at 11,5 weeks, decreased ERK phosphorylation and Wnt11 expression (both are the result of Gdnf signalling in the UB tipp).	Uchiyama Y, Sakaguchi M, Terabayashi T, et al. Kif26b, a kinesin family gene, regulates adhesion of the embryonic kidney mesenchyme. Proc Natl Acad Sci U S A 2010; 107: 9240-9245.
SALL1	Sall1 functions in the MM triggers downregulation of the stalk- specific marker Wnt9b and the canonical Wnt downstream gene Axin2 in the outgrown UB. Downregulation of Wnt9b and canonical Wnt signalling in the ureter tip is required to initiate UB branching.	Kiefer SM, Robbins L, Stumpff KM, et al. Sall1-dependent signals affect Wnt signaling and ureter tip fate to initiate kidney development. Development 2010; 137: 3099-3106.
MDM2	Mdm2 / Mdm4 deletion from the ureteric epithelium results in UB branching defects, underdeveloped nephrogenic zone with decrease in Wnt signalling from the ureteric epithelium, as a result of decreased Wnt9b expression. Deletion of Mdm2 resulted in greatly reduced or lost progenitor markers such as Sall1, Pax2, Eya1 and Bmp7. Increased p53 levels drive increased apoptosis and reduced proliferation. See even KEGG p53 signalling pathway (http://www.genome.jp/kegg/pathway.html).	El-Dahr S, Hilliard S, Saifudeen Z. Regulation of kidney development by the Mdm2/Mdm4-p53 axis. J Mol Cell Biol 2017; 9: 26-33.
WNT5, ROR	Wnt5a-Ror independent of the canonical Wnt/b-catenin-dependent pathway regulates the cellular stability of Kif26b by inducing its degradation via the ubiquitin-proteasome system, Exogenous Wnt3a also signals via ROR2 to downregulate the expression of Kif26b.	Susman MW, Karuna EP, Kunz RC, *et al.* Kinesin superfamily protein Kif26b links Wnt5a-Ror signaling to the control of cell and tissue behaviors in vertebrates. *Elife* 2017; **6**.
RET, GREB1L	RET is also a target of retinoic acid signalling via retinoic acid receptors (RARs) and GREB1L is a likely cofactor of this receptor.	Brophy PD, Rasmussen M, Parida M, et al. A Gene Implicated in Activation of Retinoic Acid Receptor Targets Is a Novel Renal Agenesis Gene in Humans. Genetics 2017; 207: 215-228.
SLIT, ROBO2	SLIT2/ROBO2 signalling is in action anterior to the normal site of UB development to prevent the formation of supernumerary UBs. SLIT2/ ROBO2 signalling is required, directly or indirectly, for posterior localization of Gdnf expression to the vicinity of the nascent UB.	Grieshammer U, Le M, Plump AS, *et al.* SLIT2-mediated ROBO2 signaling restricts kidney induction to a single site. *Dev Cell* 2004; **6**: 709-717.
GATA3, CTNN1B	β-catenin/Gata3 pathway prevents premature cell differentiation independently of its role in regulating Ret expression. Gata3 acts downstream of β-catenin, but upstream of Ret.	Grote D, Boualia SK, Souabni A, et al. Gata3 acts downstream of beta-catenin signaling to prevent ectopic metanephric kidney induction. PLoS Genet 2008; 4: e1000316.
GLI	*GLI3R* controls ureteric bud induction and outgrowth. GLI repressor acts on UB causing less branching and on NPC (nephron progenitor cell) causing less proliferation.	Blake J, Hu D, Cain JE, et al. Urogenital development in Pallister-Hall syndrome is disrupted in a cell-lineage-specific manner by constitutive expression of GLI3 repressor. Hum Mol Genet 2016; 25: 437-447.,; Hu MC, Mo R, Bhella S, et al. GLI3-dependent transcriptional repression of Gli1, Gli2 and kidney patterning genes disrupts renal morphogenesis. Development 2006; 133: 569-578.
PAX2, PAX8	*Pax2 / Pax8*-regulated *Gata3* expression is necessary for morphogenesis and guidance of the nephric duct in the developing kidney [48].	Grote D, Souabni A, Busslinger M, et al. Pax 2/8-regulated Gata 3 expression is necessary for morphogenesis and guidance of the nephric duct in the developing kidney. Development 2006; 133: 53-61.
KCP	*KCP* enhances BMP signalling and suppresses TGF-β and Activin signalling. It may delay the progression of chronic interstitial fibrosis [17].	Soofi A, Zhang P, Dressler GR. Kielin/chordin-like protein attenuates both acute and chronic renal injury. J Am Soc Nephrol 2013; 24: 897-905.
LAMC1	The *Lamc1* gene was inactivated in the developing mouse UB. Embryonic day 12.5 kidneys revealed an almost complete absence of basement membrane proteins and reduced levels of α6 integrin and FGF2. mRNA levels for fibroblast growth factor 2 (FGF2) and mediators of the GDNF/RET and WNT11 signalling pathway were also decreased [49].	Yang DH, McKee KK, Chen ZL, *et al.* Renal collecting system growth and function depend upon embryonic gamma1 laminin expression. *Development* 2011; **138:** 4535-4544.
BMP7	BMP7 is the major BMP ligand, necessary for proliferation and survival of uninduced MM. *Bmp7* deletion leads to progenitor loss and hypoplastic kidneys. *Bmp7* is expressed in the MM and UBs and maintains the nephron progenitors through the MAPK pathway while inducing differentiation through the Smad pathway [50].	Nishinakamura R, Sakaguchi M. BMP signaling and its modifiers in kidney development. Pediatr Nephrol 2014; 29: 681-686.
DSTYK	*DSTYK* is a positive regulator of ERK phosphorylation downstream of FGF-receptor activation [7].	Wu H, Xu Q, Xie J, et al. Identification of 8 Novel Mutations in Nephrogenesis-Related Genes in Chinese Han Patients with Unilateral Renal Agenesis. Am J Nephrol 2017; 46: 55-63.
CLDN3	Overexpression of *CLDN3* in vitro leads to increased matrix metalloproteinase-2 (MMP-2) activity, a protein implicated in the degradation of the extracellular matrix during embryonic development [6]. Claudin proteins in the tight junctions interact with ZO proteins and form adapters for other proteins involved in cell signalling [51]. Adherens junctions, cell adhesion by Cadherins, also send signals to the actin cytoskeleton through adapter protein β-Catenin. Slit-Robo interaction regulates actin cytoskeleton, which regulates cell migration and differentiation. For MMPs see even Relaxin signalling pathway, Pathways in cancer (http://www.genome.jp/kegg/pathway.html).	Agarwal R, D'Souza T, Morin PJ. Claudin-3 and claudin-4 expression in ovarian epithelial cells enhances invasion and is associated with increased matrix metalloproteinase-2 activity. Cancer Res 2005; 65: 7378-7385.
UPK3A, MMP	For UPK3A – MMP interaction see KEEG Bladder cancer - Reference pathway (http://www.genome.jp/kegg/pathway.html) [52].	Kihira S, Yoshida J, Kawada Y, et al. Membrane microdomain-associated uroplakin IIIa contributes to Src-dependent mechanisms of anti-apoptotic proliferation in human bladder carcinoma cells. Biol Open 2012; 1: 1024-1034.
LIF, LIFR	LIF and LIFR participate in the MAPK, PI3K/AKT, Wnt and JAK/STAT signalling pathways. See Signalling pathways regulating pluripotency of stem cells (http://www.genome.jp/kegg/pathway.html).	(http://www.genome.jp/kegg/pathway.html).
